# Barcoded DNA-Tag Reporters for Multiplex *Cis*-Regulatory Analysis

**DOI:** 10.1371/journal.pone.0035934

**Published:** 2012-04-26

**Authors:** Jongmin Nam, Eric H. Davidson

**Affiliations:** Division of Biology, California Institute of Technology, Pasadena, California, United States of America; University of Birmingham, United Kingdom

## Abstract

*Cis*-regulatory DNA sequences causally mediate patterns of gene expression, but efficient experimental analysis of these control systems has remained challenging. Here we develop a new version of “barcoded" DNA-tag reporters, “Nanotags" that permit simultaneous quantitative analysis of up to 130 distinct *cis*-regulatory modules (CRMs). The activities of these reporters are measured in single experiments by the NanoString RNA counting method and other quantitative procedures. We demonstrate the efficiency of the Nanotag method by simultaneously measuring hourly temporal activities of 126 CRMs from 46 genes in the developing sea urchin embryo, otherwise a virtually impossible task. Nanotags are also used in gene perturbation experiments to reveal *cis*-regulatory responses of many CRMs at once. Nanotag methodology can be applied to many research areas, ranging from gene regulatory networks to functional and evolutionary genomics.

## Introduction

We recently demonstrated in the sea urchin embryo that multiplexed, quantitative *cis*-regulatory analysis can be performed by use of “barcoded" DNA-tag reporters, in which the activity of each CRM in a co-injected mixture is monitored by measuring the number of transcripts produced that include its unique tag sequence [1]. Each tag reporter contains a pair of specific PCR priming sites separated by a DNA sequence common to all the vectors in the set. In our initial work the tag set contained 13 or 129 different vectors bearing tags that could be distinguished by quantitative PCR (QPCR). Individual CRMs or putative CRMs are cloned into each tag vector, the whole set mixed together, and delivered into sea urchin eggs. After their random incorporation into the embryo genomes and development to the desired stage, RNA is extracted from the embryos, and the transcripts deriving from each of the individual tag reporters are identified by using PCR primer pairs specific to each tag. The individual expression levels are normalized to the copy numbers of tags incorporated in the same embryos, as measured in their extracted genomic DNA using the same set of tag-specific QPCR primers [1], [2]. Application of the DNA-tag reporters system has improved the throughput of CRM discovery by at least one order of magnitude compared to traditional one-by-one reporter assays. However, due to the large size of current sea urchin gene regulatory networks (GRNs) [3], [4], and the ancillary requirement for multiple *cis*-regulatory analyses of various kinds, the need arose for a vastly expanded methodology for high throughput experimental CRM analysis. Here we demonstrate such a methodology, which will be applicable to a wide range of problems in addition to GRN analysis.

The NanoString nCounter platform efficiently and accurately measures expression levels of up to several hundred genes in parallel, requiring only 100∼200 ng of total RNA [5]. The NanoString nCounter system uses gene-specific probes ∼100 base pairs (bp) long, which are each uniquely labeled with a combinatorial fluorescent code. These probes are hybridized in excess with the total RNA, thus permitting the automated quantitative visualization in a scanner of each transcript species, by recognition of its particular combinatorial fluorescent code. Absolute numbers of transcripts of each species are reported by reference to standards. NanoString nCounter results are of high fidelity, and the sensitivity of the current instrumentation is at least comparable to that of QPCR. The NanoString nCounter system has been used to measure gene expression levels in GRN analysis in both sea urchin embryos and mammalian cells [3], [6], in which the numbers of genes and perturbation conditions examined far exceeded current throughput of QPCR. These studies show that the technology is optimal for system-wide assessment of the effects of specific perturbations of gene expression on large numbers of putative target genes, the experimental basis of causal GRN analysis. We decided to try to adapt NanoString technology to use with *cis*-regulatory expression vector sequence tags, and then to expand the assay system to take advantage of the highly efficient, multiplex operating principles of the NanoString nCounter. This however required complete redesign of our prior sequence tag system and of the nature of its “barcode."

## Results

### Architecture of Nanotag vectors

**Figure 1:**
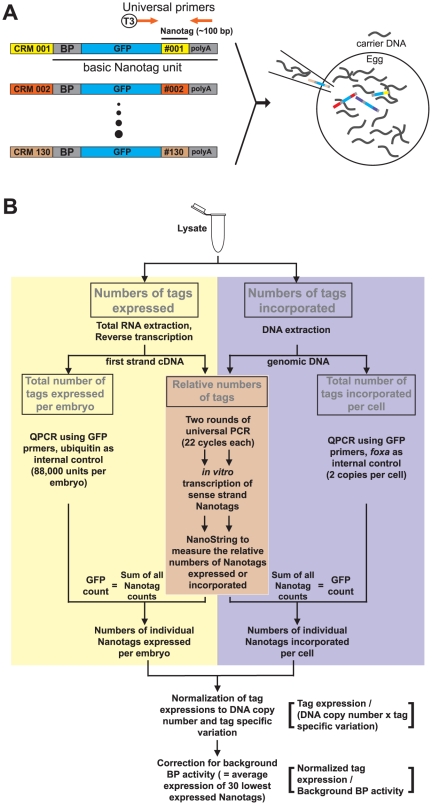
Architecture of Nanotags vectors and overview of workflow for the Nanotag-assisted *cis*-regulatory analysis. (A) Architecture of Nanotag vectors. Each Nanotag vector is composed of a basal promoter (BP) derived from *gatae* [7], a GFP open reading frame, a unique ∼100 bp-long Nanotag sequence, and a core polyadenylation signal [8]. CRM::Nanotag constructs are built, pooled, and microinjected as described in Nam et al [1]. A pair of universal primers (orange coded) is designed for signal amplification. The forward universal primer contains T3 promoter sequence in its 5′-end for *in vitro* transcription of the sense strand of the entire pool of amplified Nanotags. Figure 1A is modified from Nam et al [1]. (B) Overview of the Nanotag-assisted *cis*-regulatory analysis. Two parallel protocols respectively for measuring the numbers of individual tags expressed (left panel) and those of incorporated (right panel) are shown. While QPCR is used to measure the total number of tags expressed per embryo from first strand cDNA or the total number of tags incorporated per cell from genomic DNA, the NanoString was used to measure the relative numbers of individual tags from amplified cDNA or amplified genomic DNA with much improved sensitivity. The numbers of individual tags incorporated per embryo are estimated by comparing results from QPCR and NanoString analyses. The level of tags expressed is corrected for DNA copy number, tag specific variations, and background BP activity specific to each sample.

The Nanotag vectors share components and procedures with the earlier tag vectors [1], but newly designed Nanotag reporters have now replaced earlier QPCR-tag reporters, and other features have been added as well. The vectors into which the CRMs or putative CRMs are inserted contain a *gatae* basal promoter (BP) [7], a GFP open reading frame, a unique Nanotag sequence of about 100 bp in length that can be used for either NanoString or QPCR detection, and a core polyadenylation signal [8] (**Fig. 1A**). To increase sensitivity, we also inserted a pair of universal primer sites designed to permit PCR amplification of the entire pool of tagged sequences from either cDNA or genomic DNA. In addition, a T3 promoter sequence was added to the forward universal primer for *in vitro* transcription, since the NanoString platform is designed primarily for detecting RNA molecules. A set of 150 distinct Nanotag vectors was constructed, and a Nanotag codeset, that is, a set of combinatorially designed, fluorescent, NanoString probes uniquely complementary to the sense strands of each of the Nanotag sequences was manufactured (by NanoString, Inc.). We also provide a simple procedure for replacing the *gatae* BP with any other BP by PCR in the process of amplifying the basic Nanotag vectors (see **Methods**). The Nanotag vectors are individually fused to CRMs or to larger DNA fragments containing CRMs, and pooled for simultaneous introduction into eggs. In the sea urchin gene transfer system, linear constructs together with a seven-fold molar excess of carrier DNA, i.e., randomly sheared sea urchin genomic DNA, are injected into fertilized eggs [9]. In general, a total of about 200 molecules representing the whole mix of Nanotag expression constructs is injected into each egg. The injected linear DNAs form random concatenates, which are taken up by the embryo genomes in early cleavage stages in a mosaic fashion, and after incorporation, are replicated at the same pace as the endogenous genomes of the host cells [9], [10], [11], [12]. As before, the number of transcripts produced by each individual Nanotag vector was normalized to the number of genomically incorporated cognate tags, as measured respectively from RNA and genomic DNA in the same population of embryos [1], [2]. Gene expression (using the standard codeset designed to detect endogenous mRNAs) and CRM activities from the 130 Nanotag vectors (using the Nanotag codeset) can be reliably measured simultaneously from a starting sample of about 200 embryos. Note that a much smaller number of embryos is sufficient for good technical reproducibility, i.e., 100 embryos as in **Figure S1**.

### Overview of Nanotag-assisted CRM analysis

An overview of the Nanotag construct activity analysis protocol we have developed is shown in **Figure 1B**. The analysis consists of two parallel pathways, in one of which we measure the numbers of individual tags expressed per embryo from extracted RNA (cDNA) while in the other we measure the numbers of individual tags incorporated in the genome from extracted genomic DNA. To measure the numbers of individual tags expressed, first, the entire pool of expressed tags from first strand cDNA is amplified by two rounds of PCR using universal primers. Each round of PCR consists of 22 cycles of amplification to generate sufficient amount of PCR product for visual inspection and for *in vitro* transcription (see below). The PCR product is subsequently transcribed *in vitro* from the T3 primer, and the resulting RNA is used for NanoString detection of all 130 Nanotags. Note that any potential amplification biases specific to each tag should be common in both cDNA and genomic DNA templates, so the effect of such bias to final results should be minimal. The relative NanoString counts are proportional to the numbers of individual Nanotags expressed per embryo. Second, the total number of expressed tags per embryo of all species taken together is measured from first strand cDNA by QPCR, using GFP primers since GFP is common to all the tag vectors, plus ubiquitin primers to provide a quantitative standard (88,000 ubiquitin sequences per embryo) [13]. Third, to estimate the numbers of individual tags expressed per embryo, the sum of the NanoString counts is scaled to the number of GFP molecules expressed per embryo, and the proportion of the total counts accounted for by each tag species now gives the number of molecules of each tag vector product. To measure the numbers of individual tags incorporated per cell essentially the same protocol is used with genomic DNA as starting material, and the single copy *foxa* gene sequence is used as an internal genomic DNA standard (2 copies per cell). Because the relative incorporation of individual constructs in different samples was consistent (<20% variation) for the same pool of Nanotag constructs, NanoString analysis for genomic DNA is conducted for three samples, and the averaged profile is scaled to the total number of tags incorporated in other samples. The total numbers of tags expressed are normalized to the copy numbers incorporated for individual tags, to obtain the absolute number of each tag incorporated. There is a further small correction for the variations among Nanotags (see below). Note that potential biases during universal amplification will be cancelled out by this step. The numbers are further normalized to background activity of the BP in each sample, which is an average of the values for the 30 lowest expressed Nanotags. A known negative control, the 3-prime fragment (3P) of *nodal* [14], showed activity very close to the estimated background activity for most of the time points we tested and never showed activity higher than 2-fold of the background except at 11 hpf (2.98 fold). The background normalization is necessary, because the background BP expression is significant and increases as the embryo develops, i.e., about 2 transcripts per DNA molecule in the early blastula stage and about 5 in the mid blastula and gastrula stages. Nanotag expressions at least 2.5 fold higher than the background expression are considered significant [1], and the cumulative Poisson probabilities (P-values) of this cut off ranges from about 0.05 in the early blastula stage to ≤0.01 in later stages. The sequences of the Nanotags are available as **Data S1**.

### Variations among Nanotags

**Figure 2:**
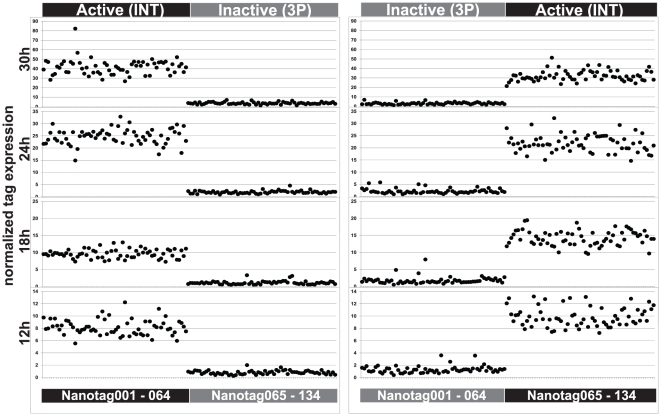
Variations among 130 Nanotags driven by the same CRM at four time points. Right panels show expression levels of the first half of Nanotag vectors driven by the same known active CRM, the *nodal* INT [14] and the other half driven by the same known inactive DNA fragment, the *nodal* 3P [14]. The Nanotag vector and driver pairs were swapped in left panels. Expressions of Nanotags were normalized to the numbers of DNA copies incorporated into the sea urchin genome. Note that four Nanotags 082, 083, 125, and 133 are missing due to problems in manufacturing probes.

To test for any intrinsic variations among the Nanotag reporters, the same known active CRM, the intron fragment (INT) of *nodal* was inserted in half the vectors while the other half received the same known inactive DNA fragment, nodal 3P [14]. The active and inactive CRM vectors were pooled and injected to provide both positive and negative controls in the same experiment. The active CRM was then inserted in the half of the vectors which had previously contained the inactive CRM, and vice versa. About 20 out 150 Nanotags initially tested showed more than two fold deviation from the average expression when driven by the active CRM, and these were removed from the set. The remaining 130 Nanotags showed only very moderate intrinsic variations (**Fig. 2**), and the pattern of variations was similar at the four time points measured, 12, 18, 24, and 30 hours post fertilization (hpf). The averages of the relative expression levels at the four time points were used to generate correction factors applied to iron out variations among tags in the following analyses (see **Methods**). Perhaps most importantly, **Figure 2** also shows that cross-regulation between co-injected active and inactive constructs does not occur to any detectable extent, which is consistent with our earlier observations [1], [14].

### Nanotag expressions compared to endogenous gene expressions

Using fusion PCR [15], [16], we then built a Nanotag vector library of 126 different DNA fragments earlier shown to possess *cis*-regulatory activity, including a negative control the *nodal* 3P module as above. The average length of these DNA fragments is about 3 kilobase pairs (kb). Most of these DNA fragments had not been studied for their spatial activities, nor had they been truncated to determine the minimum size of the active CRMs they contain. However, the set did include 40 CRMs that had earlier been investigated by careful methods and thus could serve as controls for the behavior of the Nanotag vectors containing them. The remainder of the functional data in this paper was obtained with this library. The sequences and sources of the 126 DNA fragments and the names of Nanotag vectors are to be found in **Data S2**.

**Figure 3:**
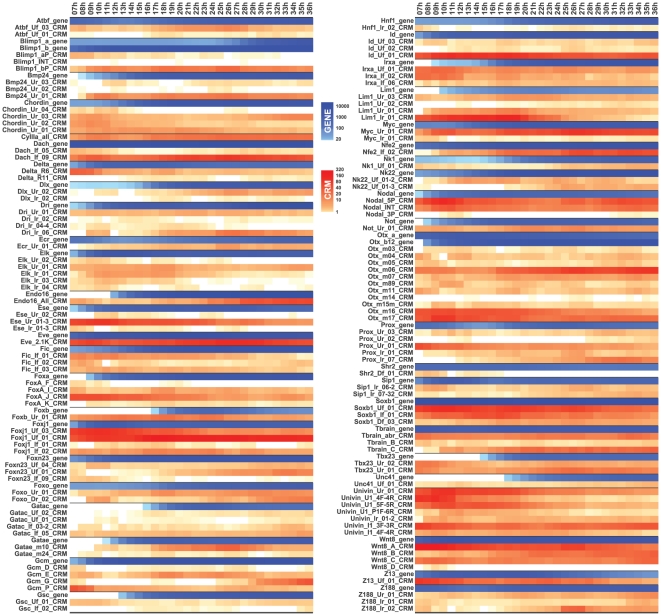
Hourly temporal profiles of CRM activities and gene expressions in developing sea urchin embryos. High resolution temporal profiles of genes and CRMs. Expression levels of the 46 genes (blue) and activities of CRMs (red) from each gene are shown in the order of 5′ to 3′ side of the gene. Temporal gene expressions were taken from Materna et al [17]. Scale of color at each time point (h) is proportional to the averages of three adjacent time points, h−1, h, and h+1. Numbers shown on the scale bar indicate the numbers of transcripts per embryo.

Recently, hourly temporal expression profiles of almost 200 genes active in the sea urchin embryo, mainly regulatory genes, were measured by Nanostring [17]. These measurements included the 46 genes to which the 126 regulatory DNA Nanotag library pertains. To compare the endogenous gene expression time courses to the behavior of the Nanotag constructs, we generated similar hourly activity profiles for the 126 constructs between 7 and 36 hpf, using 200 embryos per time point (**Fig. 3**). Simply comparing the on/off patterns, the construct activities covered >80% of the time points at which the 46 endogenous genes are expressed, and often more than one construct was active at the same or overlapping times, as we saw earlier in a smaller sample [1]. However, on closer examination, many discrepancies appeared between the activities generated by the short constructs and the endogenous genes. Occasionally, the short constructs displayed more activity than they might perform in the natural context, i.e., in the presence of the other CRMs of the gene, which might be expressed in given times and places exclusively of one another [e.g., 18]. Similarly, more than 50 of the constructs were already active at 7 hpf, the earliest time point examined, while few of the endogenous genes are expressed that early. Specifically, the nanotag constructs containing known CRMs from seven genes, *endo16*, *foxa*, *foxb*, *gatac*, *irxa*, *lim1* and *tbx2/3* showed significant activities even when these genes display no significant expression early in development. Thus the Nanotag constructs are extremely useful for fast discovery of sequences that have regulatory activity, but without further detailed examination their quantitative temporal performance cannot be taken to represent the exact regulatory output of the CRMs they contain in the natural context, i.e., multiple CRMs may functionally interact each other to generate correct gene expression and/or some CRMs may even control other neighboring genes. In addition, no evidence as to the spatial accuracy of their expression was obtained in these experiments.

### Nanotag vectors in perturbed embryos

Causal linkages in the GRN models can be validated at the *cis*-regulatory level by mutating the putative target sites of the *trans*-regulatory inputs in either short CRM constructs, or the genomic context of knock-in BAC reporters. The first step of this process, finding the relevant CRMs, is highly laborious, but this task can be enormously accelerated by use of the Nanotag system. As a demonstration project, the activities of the 126 Nanotag constructs were measured in embryos in which expression of several different genes had been alternatively perturbed, relative to normal conditions. Expression levels of about 200 sea urchin genes including the 46 genes relevant to the Nanotag library were also measured from the same samples using the NanoString nCounter system. The observed differences between control embryos and experimentally altered embryos are defined as “CRM responses" and as “gene responses" to the perturbations.

**Figure 4:**
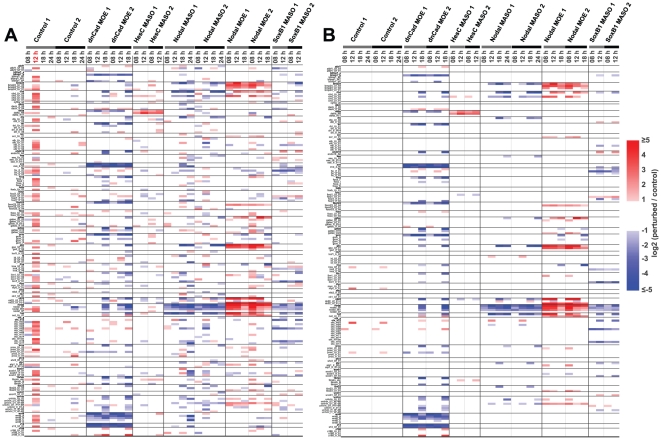
Parallel measurement of CRM responses and gene responses to gene perturbations. The order of genes and CRMs are identical to that shown in **Figure 3**. Fold change is shown in log2 scale as indicated in the scale bar. (A) Responses of the 46 genes and the 126 CRMs to five gene perturbations. Genes or CRMs that are up regulated in perturbed embryos at least two folds are red coded and those down regulated at least two folds are blue coded. All experiments were repeated in two different batches of embryos. “Control 1" is a negative control experiment, where two independent sets of control MASO (N-MASO) injected embryos in the same batch are compared, and “Control 2" is a repeat of “Control 1" with a different batch of embryos. N-MASO injected embryos in the control experiments are also used as controls for perturbed embryos. Note that any responses in the controls are false positives, and one set of the N-MASO injected embryos at 12 h (red coded time point) in “Control 1" showed overestimated activities for the majority of CRMs and is considered an outlier. (B) Reproducible responses of the 46 genes and the 126 CRMs to five gene perturbations in two batches of embryos. Only responses that were consistently observed in both experiments in **Figure 3** are color coded. All of the false positives except for five in the control experiments were not reproduced, and four reproduced false positives are due to abnormality in one of the N-MASO injected embryos at 12 h. A significant fraction of responses in perturbed embryos were reproduced.

To measure the intrinsic false positive rate (or intrinsic variation) of “CRM responses," two sets of embryos from the same batch of eggs were independently injected with a control, random morpholino antisense substituted oligonucleotide (N-MASO), and with the 126 Nanotag construct library; the experiment was then repeated with another batch of eggs (Controls 1 and 2). CRM activities of two sets of embryos from the same batch of eggs should ideally be identical, i.e., any significant differences are to be considered false positives. Applying a conventional measure of 2-fold difference as a criterion of significance, about 10% of the pairs compared showed significant false positive “CRM responses" (**Controls 1 and 2 in** **Fig. 4A**). However, the false positive results were almost never reproduced in different batches of eggs (comparison of **Controls 1 and 2 in** **Fig. 4B**). Therefore these false positives represent random experimental variation; they are not intrinsic to specific DNA fragments or specific Nanotag vectors. Variation caused by procedures after microinjection such as harvesting embryos, RNA extraction, reverse transcription, signal amplification (universal PCR amplification and *in vitro* transcription), and NanoString detection had little effect on the false positive responses (**Fig. S1**). Therefore, true responses can easily be distinguished from false responses just by repeating the experiments. Less than 2% false positive “gene responses" occur in the same sets of embryos (**Fig. S2**), suggesting that differences in endogenous regulatory states are not responsible for the false positive rate in the Nanotag CRM responses. A probable cause of the occasional false positives is opportunistic *cis*-regulatory inputs from random genomic DNA fragments in the incorporated concatenates, or from genomic regions neighboring the integration sites.

Responses of the 126 Nanotag constructs and the 46 genes to five different gene perturbations development are shown in **Figure 4A**. The five perturbations used in this demonstration experiment were: introduction of dominant negative form of *cadherin* (dnCad) mRNA over-expression (MOE) [19], [20]; *nodal* MOE; knock-down of *nodal* function by Nodal MASO [14]; knock-down of *hesc* function by HesC MASO [21]; and knock-down of *soxb1* function by SoxB1 MASO [22]. The selected target genes of these perturbation have been relatively well studied and play essential roles in sea urchin developmental GRNs. Unlike the false positives, many CRM responses were reproduced in both batches of eggs. Consistent CRM responses and gene responses are color coded in **Figure 4B**.

In all five perturbations, a total of 59 gene responses were observed out of 230 possible cases (5 perturbations×46 genes). Of these, 30 gene responses were accompanied by 40 similar CRM responses to the same gene perturbations (**Table 1**). The remaining 29 responding genes lacked any corresponding CRM responses, indicating that the initial screen failed to recover the relevant CRM, or that the CRM was truncated and suffered loss of function. There are also several cases where the Nanotag constructs responded while the endogenous genes did not. In the majority of cases, where gene and CRM responses were coordinate, the constructs are likely to contain the CRMs that control the respective responses of the genes *in vivo*. In the dataset are eight regulatory connections that had previously been validated by *cis*-regulatory evidence. Of these six were captured in Nanotag construct CRM responses to the relevant perturbations (Genes and CRMs with underlines in **Table 1**) and two were missed (Genes with asterisks in **Table 1**). The two missed connections are negative inputs into probable dedicated repressor CRMs, region F of *foxa* [23] and region 2 of *blimp1* [24]; neither of these CRMs displayed significant activity by themselves in our initial Nanotag screens and so they were missed. In conclusion, the integrated analysis of gene responses and CRM responses to gene perturbations is shown here to work as a high throughput approach to identify probable CRMs that mediate *trans*-regulatory inputs into genes. The efficiency of this method depends simply on the fraction of CRMs recovered and included in the tested library.

**Table 1:** List of genes and CRMs that showed comparable responses to the perturbations.

Perturbation	dnCad MOE (80 pg/µl)	HesC MASO (100 µM)	Nodal MASO (100 µM)	Nodal MOE (80 pg/µl)	SoxB1 MASO (100 µM)	Total
Gene (CRMs)^a^	16 (20)^b^	1 (1)	2 (3)	8 (10)	3 (6)	30 (40)
	blimp1b* (bP)^c^, chordin (U3), eve (2.1k), *ficolin (I1)*, foxa* (I), gatae (m10), gcm (E), lim1 (I1), nodal (5P, INT), *otxb1/2 (m06)*, prox1 (U1), tbrain (C), univin (U1, I1-2), wnt8 (A, B, C), z13 (U1), *z188 (I2)*	*delta (R6)*	chordin (U3), nodal (5P, INT)	*bmp2/4 (U2, U3)*, *chordin (U3)*, *gatac (U2)*, gcm (E), *gsc (U1)*, *nk2.2 (U1-3)*, *nodal (5P, INT)*, *not (U1)*	ficolin (I1, I2), nodal (5P, INT), otxb1/2 (m6, m16)	

dnCad MOE, dominant negative *cadherin* mRNA over expression; HesC MASO, translation blocking MASO against *hesc*; Nodal MASO, translation blocking MASO against *nodal*; Nodal MOE, *nodal* mRNA over expression; SoxB1 MASO, translation blocking MASO against *soxb1*. Concentration or molarity of each reagent injected is shown under the name of each reagent.

a Genes and CRMs (within parenthesis) up regulated by the perturbation are shown in italic type and those down regulated in roman type.

b Numbers of genes and CRMs (within parenthesis) responded in the perturbation are shown.

c Genes and CRMs with known *cis*-regulatory information are underlined, and genes with asterisks (*) means that we failed capturing CRMs with known *cis*-regulatory information; *blimp1b* and *eve* [24], *foxa* [23], *wnt8* [26], *delta* [27], and *nodal* [14].

## Discussion

The Nanotag vector system has many possible applications, some of which are listed in **Table 2**. Essentially this methodology enables well over a hundred *cis*-regulatory constructs to be analyzed simultaneously, in single experiments, a vast saving of time and effort. Perhaps the major general application is vastly accelerated CRM discovery and validation in genomic regions and in genes of particular interest, in the absence of any prior regulatory information. Finding CRMs is the most time consuming aspect of traditional *cis*-regulatory analysis. The multiplexed power of the Nanotag system makes prior criteria such as interspecific sequence conservation less important as a search tool, with the payoff of a more unbiased analysis. Initial screens such as we used in this work to obtain the average 3 kb fragments in the 130 construct library can now easily be followed by secondary rounds of screens in which those large fragments shown to be active are further dissected to obtain the active CRMs per se. Large sets of Nanotag CRM vectors can be used to search efficiently for the particular modules that respond to inputs predicted by GRN analysis, as in the pilot experiments of **Figure 4**. This is a common and ever present requirement in experimental GRN projects. Sometimes, particularly in the early embryo, endogenous responses are obscured by maternal transcript populations and expression constructs provide the best way seeing such responses. Although we did not capitalize on this feature in the present work, each Nanotag vector is equipped with a GFP reporter, which can be used to establish spatial activity of any CRM of interest that it contains. Alternatively, *in situ* hybridization probes could be designed against specific Nanotags. We may anticipate that a major use will be in multiplexing *cis*-regulatory analyses of mutated CRMs in order to establish the functional meaning of given transcription factor target sites; at present it is far easier and quicker to build site-specific mutations in *cis*-regulatory constructs than to test them for function, and this will now no longer be the case. The Nanotag system naturally potentiates comprehensive *cis*-regulatory analysis for whole sets of genes, whereas at present the limited number of CRMs that can be studied requires a priori choices often based on inadequate information.

**Table 2:** Possible uses of the Nanotag reporters.

Categories	Applications
**Discovery/validation of CRMs**	•Scanning of CRMs in selected regions in the genome-Overlapping, non-overlapping or nested fragments can be tested simultaneously in multiple time points.•Authentication of a large number of predicted CRMs based on sequence conservation, protein binding profiles, epigenetic signatures, or other computational analyses•Test of artificially designed DNA sequences for *cis*-regulatory activities-Different arrangements of transcription factor binding sites can be compared to study *cis*-regulatory grammar.-Different combinations/arrangements of multiple CRMs can be compared simultaneously.
**Characterization of CRMs**	•Test of CRM responses to gene perturbations-Systematic, integrated analysis of genes and CRMs is possible for fast mapping of GRNs.-Testing predicted *trans*-regulatory inputs based on computational or experimental analyses is possible.-Elucidate early regulation of genes with maternal transcripts that obscure gene expression changes.•Systematic mutational analysis of a large number of CRMs
**Internally controlled comparative ** ***cis*** **-regulatory analyses**	•*Cis*-regulatory divergence/conservation of duplicated genes•Inter-species comparison of *cis*-regulatory controls•Allelic divergence of *cis*-regulation
**Post-transcriptional regulations**	•All the above mentioned uses of the Nanotags are also applicable to *cis*-regulatory elements for post-transcriptional regulations

New technologies not only facilitate more efficient attainment of old objectives but offer opportunities for entirely new objectives as well. As **Table 2** indicates, regulatory population genetics, evolutionary problems, and other applications now come into view as well. Another whole class of experimental applications likely to be potentiated by Nanotag technology is exploration of *cis*-regulatory structure/function relations by wholly or partially synthetic means. It becomes ever easier, more inexpensive, and faster to generate DNA sequences and assemble vectors of any design, so that comprehensively designed, large sets of *cis*-regulatory variants can easily be synthetically created, in which sequence spacing, site multiplicity, site quality, site combinatorics, and other aspects of *cis*-regulatory structure are systematically varied. But the problem of testing extensive synthetic *cis*-regulatory sets in developmental contexts *in vivo*, in sufficient depth, has inhibited such projects. Nanotag technology opens the door to this sorely needed type of research. A related area of investigation that devolves from comparisons such as those in **Figures 3** and **4B** concerns the possible discrepancies not infrequently observed between the behavior of the short constructs and the regulatory behavior of the parent genes or what might be the regulatory behavior of the individual CRMs in their natural genomic context. The Nanotag system provides the opportunity to study this on a statistically large enough scale to determine whether such discrepancies, known now on an anecdotal basis [e.g.], [ 7,18], have a predictable underlying cause.

Finally, we note that the Nanotag assay system would be applicable to any model developmental system, for which efficient methods of DNA delivery have been worked out, requiring for any given application only minor modifications in the vector such as use of an endogenous basal promoter and vector backbone. Note that the Nanotag-assisted CRM analysis does not require incorporation of reporter constructs into chromosome and is compatible with transient reporter assays. The Nanotags are also compatible with QPCR and are of course freely available to scientific community.

## Methods

### Design and construction of Nanotag vectors and probes

The Nanotag sequences were selected from random sequences using the program FastPCR [25] and sequences that matched to the sea urchin genome sequence or the predicted gene set with more than 20 bp were excluded. The Nanotag vectors were built by modifying the earlier version of 129 tag vectors for QPCR detection: the earlier tag sequence was replaced with a unique Nanotag sequence by inverse PCR using primer pairs that contain each half of the new sequence at their 5-prime ends. The resulting PCR products were self-ligated and cloned. High-Fidelity Expand PCR enzyme (Roche) was used for all PCR reactions performed. Each of the new Nanotag sequences was confirmed by sequencing. A total of 150 Nanotag vectors were initially built and 130 were finally retained after a quality control experiment by QPCR. The library of NanoString probes that are complementary to the Nanotag sequences was manufactured (by NanoString Inc.). The sequences of Nanotags and vectors are provided as **Data S1**.

### Amplification of the basic Nanotag unit

We decided to switch to the *nodal* BP from *gatae* BP. There is no observed difference between the two in terms of BP activity. However, the former is much shorter (∼60 bp) and more convenient for handling than the latter (∼1.2 kb), because the latter includes a long 5-prime UTR sequence from *gatae*. Switching of the BPs is achieved during the amplification of the basic Nanotag units by PCR using new_mNBP as forward primer and T7 primer (from pGEM T-Easy backbone, Promega) as reverse primer. The sequence of the T7 primer is “5∼TAATACGACTCACTATAGGG-3′. The sequences of the new_mNBP primer is “5∼acgtcactgccagctacttcaaCTTGGAAGGTAAGGTCTCAAGTATTTAAGATTGAGGGCTCACGGGCACCTTCtcatcttacaagtgaatcacaa∼3": The sequence shown in lower characters in the 5-prime end matches to the CRM fragments amplified for the earlier QPCR-based tag vectors and serves as docking site for fusion PCR between CRMs and the basic Nanotag units; The *nodal* BP is shown in upper character; The sequence shown in lower characters in the 3-prime end matches to the tag vector and serves as priming site in PCR: 95°C for 2 min, 10 cycles (95°C for 15 s, 58°C for 30 s, 68°C for 1 min), 13 cycles (95°C for 15 s, 58°C for 30 seconds, 68°C for 1 min), 68°C for 7 min. The High Fidelity Expand Polymerase (Roche) was used in all of PCRs. The amplified basic units are column purified (ZR-96 DNA Clean-up Kit, Zymo Research) and used for fusion PCR.

### Making of CRM::Nanotag constructs

CRMs were individually amplified from genomic DNA or BAC DNA. The sequences and sources of CRMs used are provided as **Data S2**. The 5-prime end of reverse primers for the CRMs contain a sequence complementary to the 5-prime end of the amplified basic Nanotag units and serve as a docking site between each CRM and the basic Nanotag unit in fusion PCR [1], [15], [16]: 95°C for 2 min, 10 cycles (95°C for 15 s, 58°C or 60°C for 30 s, 68°C for 4 min), 15 cycles (95°C for 15 s, 58°C or 60°C for 30 s, 68°C for 4 min/+5 s per cycle), 68°C for 7 min. Fusion PCR products were column purified (ZR-96 DNA Clean-up Kit, Zymo Research), and the pairs of CRMs and Nanotags were confirmed by sequencing.

### Making and injection of CRM::Nanotag library

Equimolar amounts of CRM::Nanotags were pooled and the pooled constructs were injected as described in Nam et al [1]. The constituents of a 10 µl injection solution are 7.5 ng of CRM::Nanotags pool, 1.2 µl of 1 M KCl, and 130 ng of randomly sheared sea urchin genomic DNA as carrier. MASOs or *in vitro* transcribed mRNAs are added to the final concentration or molarity shown in **Table 1**. The intended volume for micro-injection was 2 picoliter per egg.

### Sampling and processing of embryos

About 200 laboratory grown embryos were sampled at various time points and were lysed in 20 µl lysis buffer (AllPrep DNA/RNA micro kit, Qiagen). When we needed, 4 µl of lysate was used for the direct measurement of endogenous gene expression levels by the NanoString and the remainders were used for DNA/RNA extraction. Total RNA was eluted with 15 µl of RNase-free water. Fourteen microliter of total RNA was used for reverse transcription (RT) using the iScript cDNA Synthesis Kit (Bio-Rad), and five pmole of an oligomer complementary to the 3-prime end of the basic Nanotag unit was used for more efficient RT of Nanotag transcripts. The sequence of the oligomer is “5-ATTTGTTCACGTGAG-3." One microliter of total RNA was saved for non-RT negative control QPCR with GFP primers. Reverse transcribed cDNAs were ethanol precipitated using 5 µg of glycogen as carrier and dissolved in 15 µl RNase-water. One microliter of cDNA was used for QPCR analysis for measuring the total number of Nanotags expressed per embryo by GFP primers and ubiquitin primers (internal control, 88,000 units per embryo), and delta *C*_t_ method was used for computation [13]. Ten microliter of cDNA was used for universal amplification by two rounds of PCRs: 95°C for 2 min, 10 cycles (95°C for 15 s, 58°C for 30 s, 72°C for 45 s), 12 cycles (95°C for 15 s, 58°C for 30 s, 72°C for 45 s), 72°C for 2 min. The sequences of the universal primers and QPCR primers for GFP and ubiquitin are as follows: T3_NanoTag_ampF (forward primer), 5-AATTAACCCTCACTAAAGGGAGAgatccgtgcagctggccgac-3; NanoTag_ampR (reverse primer), 5-CTTTATTTGTTCACGTGAGATCT-3; GFP_F, 5-AGGGCTATGTGCAGGAGAGA-3; GFP_R, 5-CTTGTGGCCGAGAATGTTTC-3; Ubq_F, 5-CACAGGCAAGACCATCACAC-3; Ubq_R, 5-GAGAGAGTGCGACCATCCTC-3.

Genomic DNA containing CRM::Nannotag constructs was eluted with 30 µl of RNase-free water. Two microliters of cDNA was used for QPCR analysis for measuring the total number of Nanotags incorporated per cell by GFP primers and *foxa* primers (internal control, 2 copies per cell), and delta *C*_t_ method [reviewed in 13] was used for computation. Five microliters of genomic DNA was used for universal amplification using the same set of primers and protocols mentioned above. The sequences of *foxa* primers are as follows: FoxA_F, 5-CCAACCGACTCCGTATCATC-3; FoxA_R, 5-CGTAGCTGCTCATGCTGTGT-3.

### *In vitro* transcription and NanoString analysis

About 100 ng of column purified PCR product of universal amplification was used for *in vitro* transcription (10 µl reaction volume) using T3 RNA Polymerase-Plus kit (Ambion) following manufacturer's instruction. Note that the Ambion discontinued T3 RNA polymerase, so we have switched to T7 RNA polymerase in current protocol and T7 promoter is used in the NanoTag_ampF primer. *In vitro* transcribed RNA was enthanol precipitated, and 20 pg of the RNA was used for hybridization with NanoString probes following manufacturer's instruction (NanoString).

In the perturbation experiments endogenous gene expression was measured by the NanoString. Four microliters of lysate (∼40 embryos) was directly applied to hybridization following manufacturer's instruction (NanoString Inc.). Count of ubiquitin was used as internal control.

### Data analysis

Analysis of Nanotag data was conducted in the following order: 1) The NanoString counts of individual Nanotags expressed and those of incorporated were respectively scaled to the number of GFP transcripts and that of incorporated measured by QPCR. 2) The numbers of Nanotags expressed were normalized to the numbers of Nanotags incorporated. 3) The normalized Nanotag expression was corrected for variation among tags, which is provided as **Data S3**. 4) Normalized Nanotag expression was corrected to the background BP activity specific to each sample, which is an average of the 30 lowest expressed Nanotags. Note that background corrected level of the Nanotag driven by a negative control fragment, *nodal* 3P, was about 1 in almost all of the samples we analyzed. As in the case of our earlier study, Nanotag expressions at least 2.5 fold higher than the background activity are considered significant in this study. 5) To compute fold changes of CRM activities in the perturbation experiments, the following analytical criteria were applied: Background corrected CRM activity lower than 1 was treated as 1; For a pair of CRM activities compared at least one CRM should have background corrected activity of 2.5 or higher; Any pairs that did not match these criteria were considered insignificant. 6) CRM responses consistent in repeat experiments were considered true responses.

Analysis of gene expression data from perturbation experiments was conducted in the following order: 1) The levels of gene expression in each sample were normalized to the number of ubiquitin units, 88,000 units per embryo. 2) Estimated copy number of transcripts per embryo higher than 20 was considered significant. 3) For a pair of gene expression levels compared at least one gene expression should be higher than 20 copies per embryos, otherwise the change was considered insignificant.

To compare hourly CRM activity profiles with hourly gene expression profiles we used the averages of gene expression levels of replicate experiments in Materna et al [17].

No specific permits were required for the described field studies.

## Supporting Information

Figure S1 **Highly reproducible technical replicates of the Nanotag procedure from two independently sampled populations of embryos at 24 h from the same injection.** Universal amplification and *in vitro* transcription were performed prior to NanoString analysis. About 100 embryos injected with the 126 Nanotag constructs were sampled for each replicate. There was no Nanotags that showed more than 2-fold differences and most of the Nanotags showed less than 30% variations. We did not test other time points, as this experiment was for testing technical reproducibility rather than biological reproducibility.
(EPS)

Figure S2 **Variations of the expression of 212 genes in the same control samples as shown in** **Figure 4** **.** Gene expressions showed much smaller variations than CRM activities, and there was no obvious correlation between variations in gene expressions and CRM activities.
(EPS)

Data S1 **The sequences of the Nanotags.**
(XLSX)

Data S2 **The sequences and sources of the 126 DNA fragments and the names of Nanotag vectors.**
(XLSX)

Data S3 **Variation among Nanotags.**
(XLSX)
